# Downregulating p22phox ameliorates inflammatory response in Angiotensin II-induced oxidative stress by regulating MAPK and NF-κB pathways in ARPE-19 cells

**DOI:** 10.1038/srep14362

**Published:** 2015-09-29

**Authors:** Yiguo Qiu, Lifei Tao, Chunyan Lei, Jiaming Wang, Peizeng Yang, Qiuhong Li, Bo Lei

**Affiliations:** 1 Department of Ophthalmology, the First Affiliated Hospital of Chongqing Medical University, Chongqing Key Laboratory of Ophthalmology, Chongqing Eye Institute, Chongqing, China; 2 Department of Ophthalmology, University of Florida, Gainesville, Florida, USA

## Abstract

Oxidative stress and inflammation are two interrelated biological events implicated in the pathogenesis of many diseases. Reactive oxygen species (ROS) produced under oxidative stress play a key role in pathological conditions. Inhibition of p22phox, an indispensable component of the NADPH oxidase (NOX) complex comprising the main source of ROS, plays a protective role in many ocular conditions by inhibiting the activation of NOXs and the generation of ROS. However, little is understood regarding the role of p22phox in oxidative stress-related inflammation in the eye. We used a p22phox small interfering RNA (siRNA) to transfect the retinal pigment epithelium (RPE)-derived cell line ARPE-19, and human primary RPE (hRPE) cells, then stimulated with Ang II. We observed a potent anti-inflammatory effect and studied the underlying mechanism. Downregulating p22phox resulted in decreased ROS generation, a reduction of NOXs (NOX1, 2, 4) and a decrease in inflammatory cytokine. In addition, p22phox downregulation reduced the activation of the MAPK and NF-κB signaling pathways. We conclude that inhibition of p22phox has an anti-inflammatory effect in Ang II-induced oxidative stress. Suppressing the MAPK and NF-κB pathways is involved in this protective effect. These results suggest that p22phox may provide a promising therapeutic target for oxidative stress-induced ocular inflammation

Oxidative stress and inflammation are two interrelated biological events implicated in the pathogenesis of many diseases. Reactive oxygen species (ROS) are noxious substances largely produced under oxidative stress. Increasing evidence indicates ROS are associated with many devastating ocular diseases in which oxidative damage and inflammation are the underling pathological processes, including glaucoma^1^, age-related macular degeneration (AMD)^2^ and diabetic retinopathy^3^. Accumulating evidence has demonstrated that ROS generation correlates with inflammation. There is a vicious cycle in which oxidative stress triggers inflammatory responses, and inflammation in turn enhances the production of ROS^4^,^5^. It has been demonstrated that oxidative stress induced the release of inflammatory molecules, such as interleukin (IL)-8 and nitric oxide (NO), from macrophages and alveolar epithelial cells^6^,^7^. Similarly, retinal pigment epithelia (RPE) produce higher levels of inflammatory cytokines, such as tumor necrosis factor (TNF)-α and cyclooxygenase (COX)-2, under oxidative stress^8^.

Nicotinamide adenine dinucleotide phosphate (NADPH) oxidase (NOX) is the best known non-mitochondrial source of ROS. It is the only enzyme system that mediates ROS generation not merely as a by-product, but as its primary function^9^. NOX is an essential enzyme in phagocytes as it catalyzes ROS generation for host defense^10^. It is responsible for ROS production in various non-phagocytic cells as well^11^,^12^. NOX plays an essential role in several pathological conditions with oxidative stress and inflammation, including those of the vasculature and central nervous system^13^,^14^,^15^.

The active NOX complex consists of cytochrome b558 and three other cytoplasmic proteins. Cytochrome b558 is composed of two membrane-bound catalytic subunits, p22*phox* and gp91*phox* (also known as NOX2). The other cytoplasmic proteins include p40*phox*, p47*phox* and p67*pho,* which produce superoxide^16^. The catalytic subunits of NOX commonly present as different homologues in mammalian cells, including NOX1-5 and thyroid-specific DUOX1 and DUOX2^10^. Seven homologues of NOX exist in various tissues and cell types^10^. Previous studies suggest that NOX is involved in some retinal pathological processes. Inhibition of NOX1 and NOX2 suppressed retinal neovascularization, decreased the production of inflammatory factors and the generation of ROS, and alleviated vascular leakage in oxygen-induced retinopathy (OIR) and diabetic retinopathy^17^,^18^. However, in the aforementioned studies, the extent of the role played by NOX is unclear. Additionally, the precise function of different NOX homologues in these pathological conditions is unknown. An essential subunit of NOX that is likely to play an important role in disease pathogenesis is p22phox.

As an indispensable part of the NOX complex, the p22phox subunit is required for activating, stabilizing and/or regulating NOX homologues, with the exception of NOX5 and DUOX1/2^10^,^19^. p22phox forms heterodimers with various NOX homologues and cytoplasmic components to become stable complexes that participate in various cellular functions and contribute to diverse physiological events^10^. p22phox is found throughout the retina and is especially abundantly in inner retinal neurons and retinal pigmented epithelial cells^20^. A recent study proposed that modulation of p22phox may interfere in the common ocular pathological condition, choroidal neovascularization (CNV). Downregulation of p22phox by AAV-mediated delivery of small interfering RNA (siRNA) dramatically inhibits the development of CNV^20^. Knockdown of p22phox decreased cell proliferation in human lens epithelial cells by reducing NOX activity and regulating the activation of the mitogen-activated protein kinase (MAPK) and Akt signaling pathways^21^. Accordingly, p22phox could be a straightforward target for breaking the cycle of oxidative damage and inflammation in various pathological events characterized by oxidative stress-related inflammation.

Angiotensin II (Ang II) is the primary effector of the rennin-angiotensin system (RAS). It plays an essential part in the inflammatory process by increasing the expression of proinflammatory cytokines and chemokines^22^. The majority of intracellular actions induced by Ang II are mediated by the activation of MAPK and nuclear factor-κB (NF-κB), which play vital roles in regulating proinflammatory genes at the transcriptional level^23^,^24^,^25^. Additionally, Ang II reportedly induces oxidative stress by elevating the expression of p22phox in the retina^26^.

The RPE plays an essential role in maintaining normal visual function. RPE is associated with greater oxygen consumption compared with other tissues and is primed for the production of oxidants and free radicals^27^. In addition, phagocytosis of photoreceptor outer segments by RPE cells, a critical process in visual function, also results in the generation of ROS. Hence, RPE cells are particularly vulnerable to ROS insults^27^. In response to proinflammatory stimuli, RPE are capable of producing a variety of cytokines, including IL-6, IL-8 and monocyte chemotactic protein-1(MCP-1), which are crucial to ocular inflammation^28^. The human RPE-derived cell line ARPE-19 used in this study has structural and functional characteristics of RPE cells^29^,^30^.

We hypothesize that downregulation of p22phox leads to an anti-inflammatory effect on Ang II-induced oxidative stress, with the beneficial effect being mediated by MAPK and NF-κB pathway inhibition. We used a p22phox siRNA to test the effect of downregulating p22phox on decreasing the activation of NOXs and the production of inflammatory cytokines.

## Results

### Different concentrations of Angiotensin II (Ang II) did not affect viability or apoptosis of ARPE-19 cells

**Figure 1:**
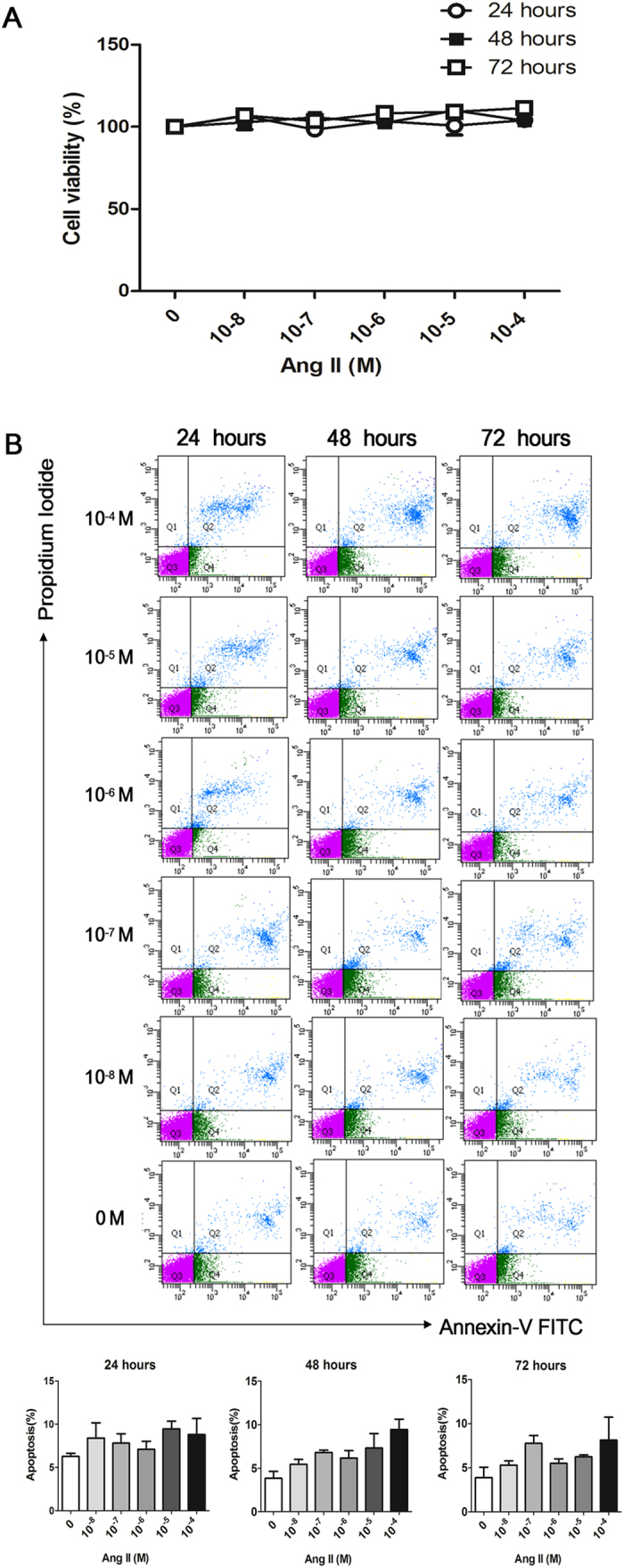
Cell viability and apoptosis of ARPE-19 cells cultured with Angiotensin II as measured with CCK-8 and Annexin-V FITC/PI assay. (**A**) ARPE-19 cells were cultured with various concentrations (10^−8^, 10^−7^, 10^−6^, 10^−5^ and 10^−4^ M) of Ang II for 24, 48 and 72 hours. Incubation with Ang II did not affect the viability of ARPE-19 cells compared with cells cultured without Ang II. Viability of each treatment group was measured compared to cells cultured without Ang II (0 M). There was no significant difference in cellular viability among the treatment groups at any time point. The data were normalized to the control group (0 M) (*p* > 0.05, n = 3). (**B**) The lower left quadrant (Q3) represents viable cells (Annexin-FITC and PI negative). The upper right quadrant (Q2) represents late apoptotic cells (Annexin-FITC and PI positive). There was no significant difference of cell apoptosis among the cells cultured with various concentrations of Ang II for 24, 48 and 72 hours. The data were shown as mean ± SEM (*p* > 0.05, n = 4).

Confluent ARPE-19 cells were cultured with different concentrations of Angiotensin II (Ang II) to determine the effect of Ang II on cellular viability and apoptosis. Incubation with Ang II for 24, 48 and 72 hours at concentrations of 10^−4^, 10^−5^, 10^−6^, 10^−7^ and 10^−8^ M did not affect the viability or apoptosis-rate of ARPE-19 cells compared with the cells cultured without Ang II (*p* > 0.05) (Fig. 1A). There was no significant difference in cell apoptosis in the cells treated with Ang II at the concentrations of 10^−4^, 10^−5^, 10^−6^, 10^−7^ and 10^−8^ M for 24, 48 and 72 hours (*p* > 0.05) (Fig. 1B). Therefore, to provide the best balance between inflammatory response and cell toxicity, the concentration of 10^−6^ M was used for subsequent experiments.

### Transfecting ARPE-19 and human primary retinal pigment epithelium (hRPE) cells with p22phox siRNA-1 (P-1) decreased the expression of p22phox at both the mRNA and protein levels

**Figure 2:**
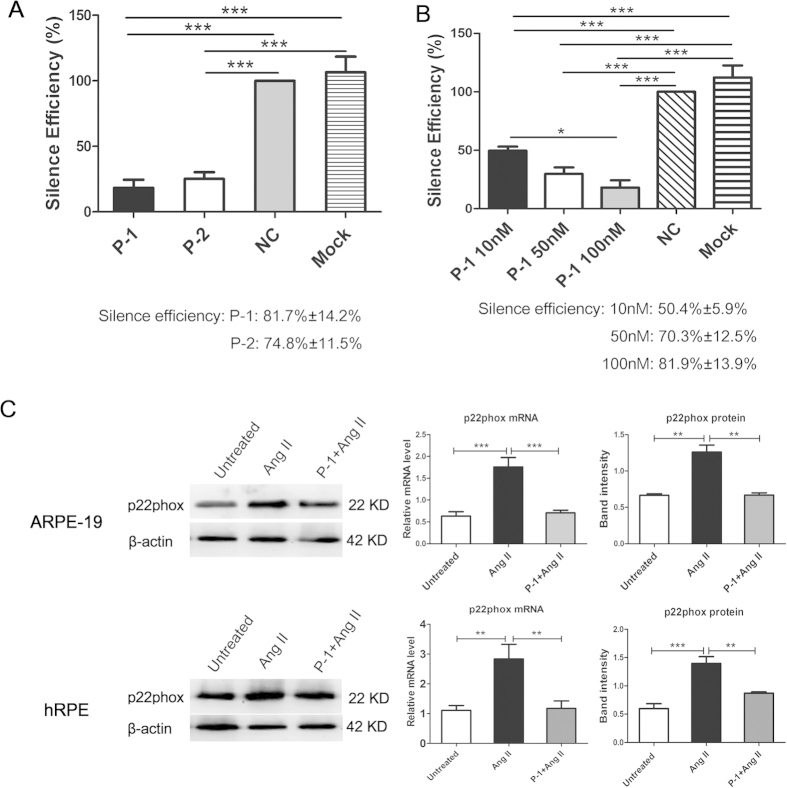
Downregulating effect of p22phox siRNA-1(P-1) on both mRNA and protein levels in ARPE-19 cells and human primary retinal pigment epithelium (hRPE). (**A**) The mRNA level of p22phox in ARPE-19 cells transfected with 100 nM p22phox siRNA-1 (P-1) and 100 nM p22phox siRNA-2 (P-2) compared with the NC group (negative control: cells transfected with scrambled siRNA) and mock transfected group (cells treated with transfection agent only) (***p < 0.001, n = 5). (**B**) The level of p22phox mRNA in ARPE-19 cells transfected with P-1 (10 nM, 50 nM and 100 nM) compared with the NC group and mock transfected group (***p < 0.001, n = 5). (**C**) The expression of p22phox in the P-1+Ang II, Ang II treated and untreated groups was detected by real-time PCR and Western blotting in ARPE-19 cells and hRPE. The expression of p22phox was remarkably lower in the P-1+ Ang II group than in the Ang II group at both the mRNA (n = 6) and protein levels (n = 3). The relative expression of mRNA and protein was normalized to β-actin. The data were shown as mean ± SEM (**p < 0.01, ***p < 0.001).

To determine the p22phox siRNA sequence with optimal silencing efficiency, both p22phox siRNA sequence-1 (P-1) and p22phox siRNA sequence-2 (P-2) were tested. ARPE-19 cells were transfected with 100 nM P-1 or P-2. Total RNA was extracted 48 hours after transfection. The silencing efficiency of P-1 and P-2 was determined by real-time PCR. The results showed that at 100 nM, both P-1 and P-2 significantly downregulated the expression of p22phox compared with that of the NC group (negative control: cells transfected with scrambled siRNA) and the mock transfected group (cells treated with transfection agent only) (*p* < 0.001). The silencing efficiency of P-1 and P-2 was 81.7% ± 14.2% and 74.8% ± 11.5%, respectively. Therefore, P-1 was chosen for subsequent experiments (Fig. 2A). Next, we determined the optimal concentration of P-1 in ARPE-19 cells. The mRNA level of p22phox was significantly decreased by transfection with P-1 at the concentrations of 10 nM, 50 nM and 100 nM (compared with the NC group and mock transfected group; *p* < 0.001). The expression of p22phox in the 100 nM group was remarkably lower than the 10 nM group (*p*<0.05). The silencing efficiency was 50.4% ± 5.9%, 70.3% ± 12.5% and 81.9% ± 13.9% for 10 nM, 50 nM and 100 nM concentrations of P-1, respectively. Thus, 100 nM of P-1 was used in subsequent studies (Fig. 2B). The expression of p22phox mRNA and protein expression were significantly increased in ARPE-19 and hRPE cells incubated with Ang II for 48 hours. However, the levels of p22phox mRNA and protein were remarkably decreased in the cells transfected with P-1 and incubated with Ang II (P-1 + Ang II group) compared with the Ang II group in both ARPE-19 and hRPE cells (*p* < 0.01, *p* < 0.001) (Fig. 2C).

### Downregulating p22phox decreased the activation of NOXs (NOX 1, NOX2 and NOX4) and the generation of intracellular ROS in Ang II-stimulated ARPE-19 and hRPE cells

**Figure 3:**
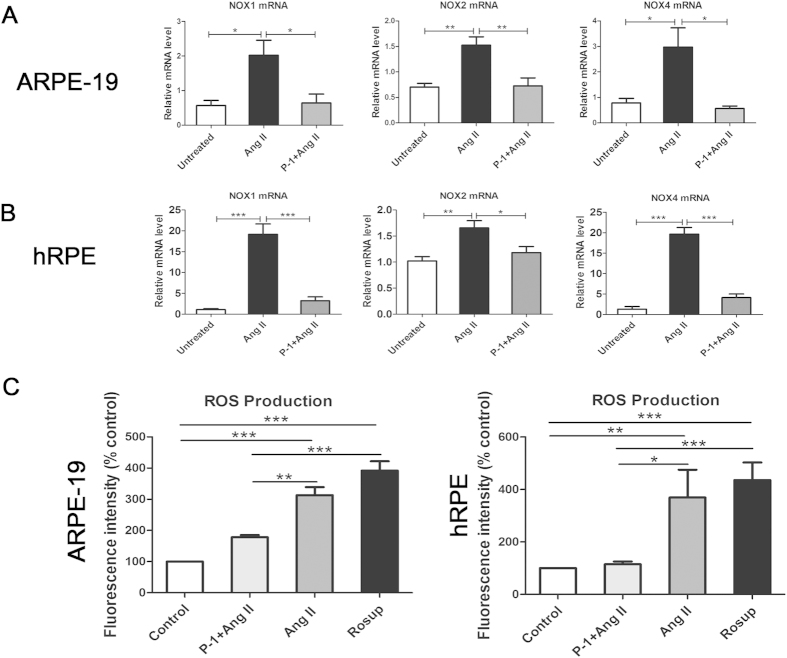
Effect of downregulating p22phox on the activation of NOXs (NOX1, NOX2 and NOX4) and intracellular ROS generation in Ang II-stimulated ARPE-19 cells and hRPE. (**A**) ARPE-19 cells were transfected with or without 100 nM P-1 in the presence or absence of 10^−6^ M Ang II for 48 h. Next, NOX1, NOX2 and NOX4 mRNA were detected with real-time PCR (*p < 0.05, **p < 0.01, n = 4–6). (**B**) hRPE were transfected with or without 100 nM P-1 in the presence or absence of 10^−6^ M Ang II for 48 h. The levels of NOX1, NOX2 and NOX4 mRNA were detected with real time PCR (*p < 0.05, **p < 0.01, ***p < 0.001, n = 4–6). (**C**) The ROS generation in each group of ARPE-19 cells and hRPE was analyzed by flow cytometry. The percentage of ROS generation is indicated on the ordinate and is normalized to the value of the control group (*p < 0.05, **p < 0.01, ***p < 0.001, n = 6–8). The data were shown as mean ± SEM.

The transmembrane protein p22phox was identified as one of the membrane-bound catalytic subunits of active NADPH oxidase complex associated with NOX2 (also known as gp91phox). As mentioned above, there are seven homologues of NOX, including NOX1-5 and the thyroid-specific DUOX1 and DUOX2. However, p22phox is required for activation and stabilization of all NOX proteins, except for NOX5 and DUOX1/2^31^. Moreover, NOX3 is exclusively expressed in the inner ear and fetal tissue^32^. Hence, we investigated the mRNA expression of NOX1, NOX2 and NOX4, as those are the NOXs that require p22phox for activation and stabilization. Downregulating p22phox remarkably inhibited the activation of NOX1, NOX2 and NOX4. The levels of NOX1, NOX2 and NOX4 mRNA were reduced in the P-1+ Ang II group compared with the Ang II treated group in ARPE-19 cells (*p* < 0.05, *p* < 0.01) (Fig. 3A) and hRPE cells (*p* < 0.05, *p* < 0.01, *p* < 0.001) (Fig. 3B). We also investigated whether downregulating p22phox inhibited ROS generation in ARPE-19 and hRPE cells exposed to Ang II. Fig. 3C showed downregulating p22phox resulted in a significant reduction of ROS production in the P-1 + Ang II group versus the Ang II treated group in both ARPE-19 and hRPE cells (control: untreated cells as the negative control. Rosup: cells treated with the ROS stimulating agent from the kit as the positive control, *p* < 0.05, *p* < 0.01, *p* < 0.001). These results indicate that downregulating p22phox inhibits Ang II-induced ROS generation, associated with the suppression of NOX.

### Downregulating p22phox decreased the expression of inflammatory cytokines at both the mRNA and protein levels

**Figure 4:**
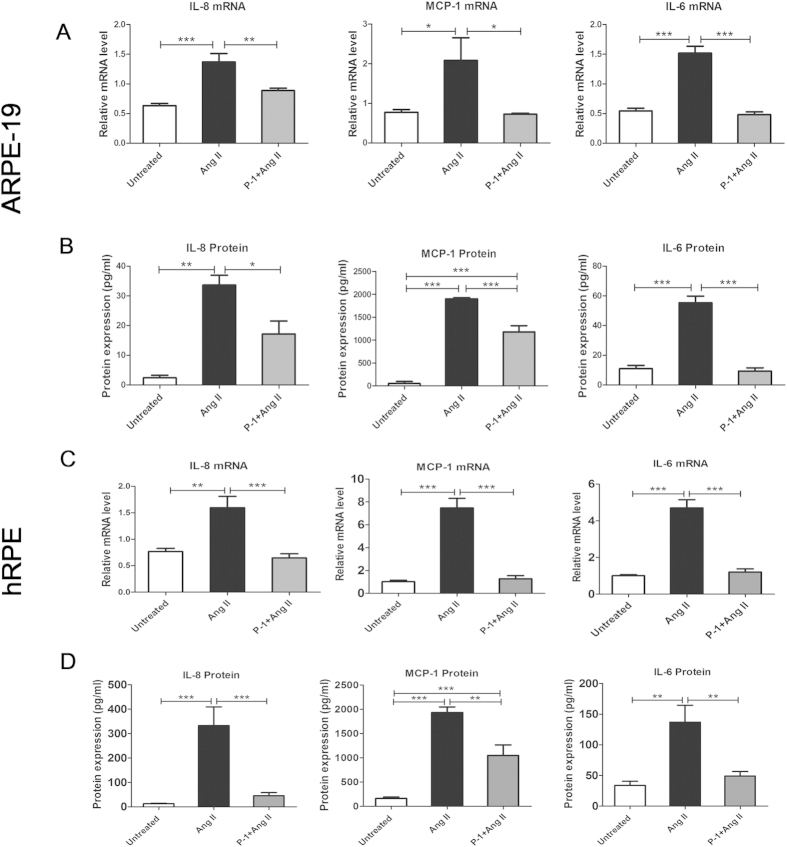
Effect of downregulating p22phox on mRNA expression and protein levels of inflammatory cytokines in ARPE-19 cells and hRPE. Total RNA was isolated from the P-1+ Ang II, Ang II and untreated control groups to analyze the mRNA levels of the inflammatory cytokines IL-8, MCP-1, IL-6 in ARPE-19 cells (**A**) and in hRPE (**C**). Cell supernatants were collected for ELISA to detect the protein concentrations of IL-8, MCP-1 and IL-6 in ARPE-19 cells (**B**) and in hRPE (**D**). The expressions of IL-8, MCP-1 and IL-6 were significantly decreased in the P-1+ Ang II group compared with the Ang II group at both the mRNA (n = 6) and protein levels (n = 4–6) (*p<0.05, **p<0.01, ***p<0.001). The data were shown as mean ± SEM.

Ang II is a potent pro-inflammatory factor that induces the production of numerous cytokines and chemokines in various types of cells and organs. Ang II induced the production of (MCP-1), IL-6 and IL-8 in monocytes, macrophages, vascular smooth muscle cell (VSMCs) and endothelial cells^33^,^34^. Furthermore, induction of MCP-1 and IL-6 by Ang II was dependent on the activation of NOX in VSMCs^35^,^36^. In accordance with the former studies, we showed that the treatment with Ang II significantly induced mRNA and protein expression of IL-8, MCP-1 and IL-6. Notably, mRNA and protein expression of IL-8, MCP-1 and IL-6 was decreased in the P-1+ Ang II group compared with the Ang II group in ARPE-19 and hRPE cells (*p* < 0.05, *p* < 0.01, *p* < 0.001) (Fig. 4).

### The MAPK and NF-κB pathways were involved in cytokine production in the Ang II-induced inflammatory response

**Figure 5:**
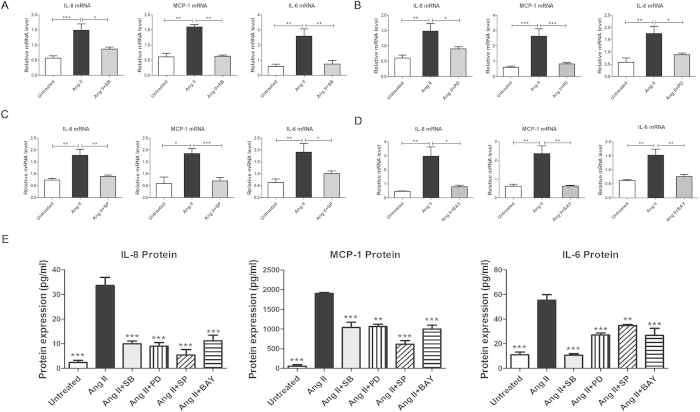
The expression of inflammatory cytokines in response to Ang II with or without the presence of MAPK and NF-κB inhibitors. mRNA expression of IL-8, MCP-1 and IL-6 was measured by real-time PCR 48 hours after stimulation by 10^−6^ M Ang II with or without the presence of the p38 MAPK inhibitor SB203580 (**A**) ERK1/2 inhibitor PD98059 (**B**) JNK inhibitor SP600125 (**C**) and NF-κB inhibitor BAY11-7082 (**D**) all at a final concentration of 10 μM. (**E**) The protein expression levels of IL-8, MCP-1 and IL-6 were measured by ELISA 48 hours after stimulation by 10^−6^ M Ang II with or without the presence of the p38 MAPK inhibitor SB203580, ERK1/2 inhibitor PD98059, JNK inhibitor SP600125 and NF-κB inhibitor BAY11-7082, all at a final concentration of 10 μM. The data were shown as mean ± SEM (*p < 0.05, **p < 0.01, ***p < 0.001, n = 4–6).

To elucidate whether the MAPK and NF-κB pathways are involved in Ang II -induced inflammatory cytokine production, ARPE-19 cells were pretreated with MAPK and NF-κB pathway inhibitors, followed by Ang II incubation for 48 hours. Inhibition of p38 MAPK with the highly selective inhibitor SB20358046 reduced Ang II-mediated production of IL-8, MCP-1 and IL-6 mRNA levels by approximately 1.7-, 2.5- and 3.5-fold, respectively (Fig. 5A); protein levels were decreased by 3.4-, 1.8- and 5.2-fold, respectively (Fig. 5E). Inhibition of ERK 1/2 with the inhibitor PD98059 reduced IL-8, MCP-1 and IL-6 mRNA levels by approximately 1.6-, 3.2- and 2.0-fold, respectively (Fig. 5B); protein levels were reduced by approximately 3.7-, 1.8- and 2.5-fold, respectively (Fig. 5E). A JNK inhibitor, SP600125, decreased mRNA expression of IL-8, MCP-1 and IL-6 by 2.0-, 2.6- and 1.9-fold (Fig. 5C), respectively; protein levels were reduced by 6.2-, 3.1- and 1.6-fold, respectively (Fig. 5E). The NF-κB inhibitor BAY11-7082 resulted in 3.7-, 3.7- and 2-fold reductions of IL-8, MCP-1 and IL-6, respectively, at the mRNA level (Fig. 5D); protein levels of the three cytokines were decreased by 3.0-, 1.9- and 2.0-fold, respectively (Fig. 5E) (p < 0.05, *p* < 0.01, *p* < 0.001). Fold changes of mRNA and protein levels are shown in Table 1.

### The protective effect of downregulating p22phox in response to Ang II stimulation is associated with MAPK and NF-κB pathways inhibition

**Figure 6:**
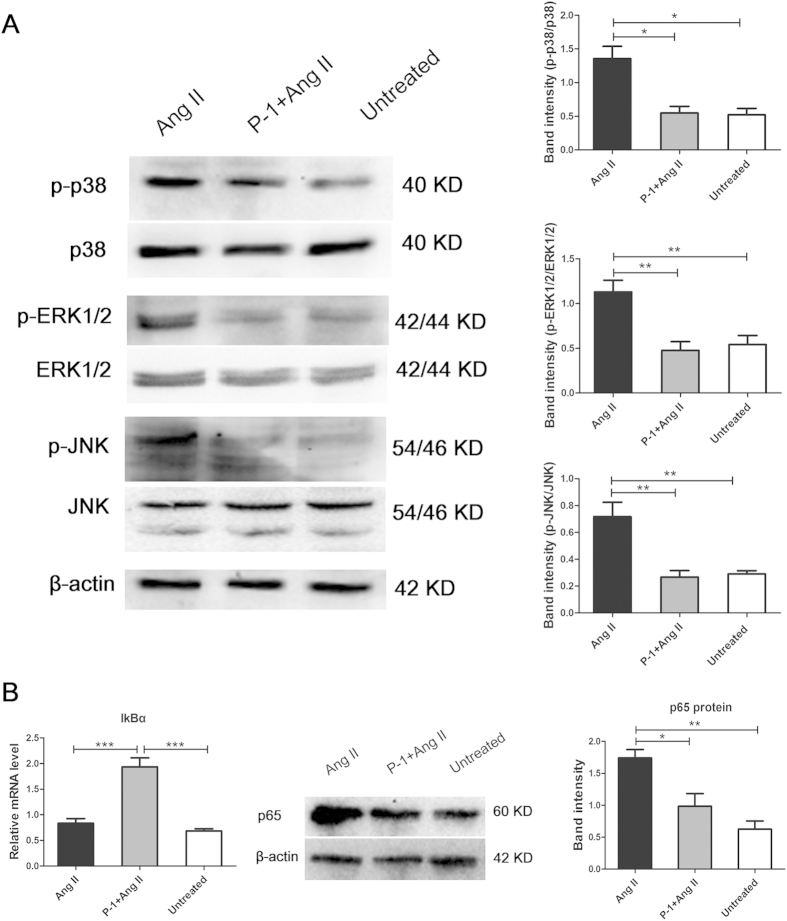
Involvement of the MAPK and NF-κB pathways in downregulating p22phox responded to the stimulation of Ang II. (**A**) The protein expression of p38 MAPK, ERK1/2 and JNK, and their phosphorylation level (p-p38 MAPK, p-ERK1/2 and p-JNK) in the Ang II group compared with the P-1+ Ang II and untreated control groups were determined by Western blotting (*p < 0.05, **p < 0.01, n = 3–6). The band intensities of p-p38 MAPK, p-ERK1/2 and p-JNK were normalized to p38 MAPK, ERK1/2 and JNK, respectively. **(B)** The mRNA expression of IκBα, the inhibitor of NF-κB, in the Ang II group compared with the P-1+ Ang II and untreated control groups was detected by real-time PCR (***p < 0.001, n = 6). The protein expression of the NF-κB subunit p65 in the Ang II group compared with the P-1+ Ang II and untreated control groups was determined by Western blotting. Relative expression was normalized to β-actin (*p <0.05, **p <0.01, n = 3). The data were shown as mean ± SEM.

To further determine whether the inhibition of the MAPK and NF-κB pathways is involved in the protective role played by downregulating p22phox in the Ang II-induced inflammatory response, ARPE-19 cells transfected with or without p22phox siRNA were incubated with 10^−6^ M Ang II for 48 hours. Changes in the phosphorylation level of p38 MAPK, ERK1/2 and JNK were measured by Western blotting. The results indicate that Ang II stimulation increased the phosphorylation of p38 MAPK, ERK1/2 and JNK. The phosphorylation levels of all the three proteins were significantly reduced in p22phox siRNA transfected cells compared to the Ang II-stimulated group (*p* < 0.05, *p* < 0.01) (Fig. 6A). Furthermore, we investigated whether the protective effect of downregulating p22phox was associated with inhibiting the NF-κB pathway. mRNA expression of IκBα, the inhibitor of NF-κB, was remarkably increased by transfection with P-1 in response to Ang II compared with the Ang II group (*p* < 0.001). The protein expression of the NF-κB subunit p65 was determined with Western blotting. P-1 transfection downregulated the transcriptional activity of nuclear p65 in Ang II-induced inflammation in ARPE-19 cells (*p* < 0.01, *p* < 0.05) (Fig. 6B).

## Discussion

In the present study, we demonstrate that inhibiting p22phox ameliorated the inflammatory response accompanying oxidative stress. Downregulating p22phox results in a decrease of intracellular generation of ROS, activation of NOXs, and the production of inflammatory cytokines in ARPE-19 and hRPE cells. Our study also indicates that phosphorylation of MAPK components and the activation of the NF-κB pathway were associated with the pathological condition accompanying the inflammatory response in ARPE-19 cells. Downregulating p22phox inhibited activation of the MAPK and NF-κB pathways.

NOXs are expressed ubiquitously in many organs and tissues. Although NOX1, NOX2, NOX4 and NOX5 are found in the human retina, the detailed distribution profiles of NOX isoforms in the retina remains unclear^17^. Phagocytosis of the shed tips of photoreceptors by the RPE is accompanied by an intracellular respiratory burst through NADPH oxidases, which suggests the involvement of NADPH oxidases and their expression in the RPE^10^,^37^,^38^. Although it is uncertain which specific NOX isoforms are expressed in the retinal pigment epithelium, here we demonstrated that NOX1, NOX2 and NOX4 were expressed in ARPE-19 cells. In addition, we found the expression of NOX1, NOX2 and NOX4 can be regulated by p22phox. A previous study showed that p22phox was mainly expressed in retinal pigmented epithelial cells^20^. Our results support the concept that p22phox localization and expression is important to NOX isoforms, which are co-expressed with p22phox in a given cell type^39^. In addition, our data support the previously held belief that p22phox forms heterodimers with various NOX isoforms. Only when the NOXs and p22phox protein form a heterodimer do they become stable and have normal function^40^.

Oxidative stress and inflammation are the two most common features shared by many diseases. Superoxide produced by the NOX complex is a potential contributor to transcriptional signal transduction pathways as well as a potential participant in the inflammatory processes^41^. The NOX complex is involved in several pathological conditions with oxidative stress and inflammation. In the vasculature, pharmacological inhibition or gene knockdown of NOX4 reversed or prevented vascular inflammation and remodeling^13^,^14^. NOX 4 inhibitors also prevented oxidative stress and the inflammatory responses in cultured cells^42^,^43^. In the central nervous system, over activation of NOX2 has been recognized as a major mediator of inflammatory oxidative neurodegeneration. Pharmacological inhibition of NOX2 activity provides a neuroprotective effect in models of neurodegenerative diseases^15^.

Recent studies have linked NOXs to retinopathy^44^,^45^. Firstly, high level of NOX1 in retinal ganglion cells (RGCs) could be a major source of ROS in RGCs under ischemic conditions^46^. Secondly, NOX is also associated with neovascularization, a common pathological process in many retinal vascular diseases. Inhibition of NOXs prevents retinal neovascularization in oxygen-induced retinopathy (OIR)^47^. A recent study showed NOX4 was increased in an OIR rat model. Knockdown of NOX4 by siRNA transfection inhibited basal and VEGF-induced ROS generation and cell proliferation in human retinal microvascular endothelial cells (hRMVECs)^48^. Similarly, NOX1 knockout mice with OIR were protected from retinal neovascularization and an increase of pro-inflammatory factors, as well as ROS^17^. Finally, inhibition of NOX alleviates vascular leakage in diabetic retina^49^. Administration of a NOX inhibitor or deletion of NOX2 suppressed an increase of retinal intracellular adhesion molecule (ICAM)-1, leukocyte adhesion and vascular permeability in an STZ-induced mouse model of diabetes^18^.

Nevertheless, it is unclear what precise functions the NOXs have, and which subunit of the NOX complex plays the predominant role in retinal disorders. It is notable that p22phox is a subunit of the NOX complex required for the activation of many NOXs^10^. In agreement with a previous study showing downregulation of p22phox mRNA alone suppressed the expression of multiple NOX isoforms and the activity of the NOX complex^50^, our results showed that transfection with p22phox siRNA inhibited the expression of NOX1, NOX2 and NOX4. Hence, the subunit p22phox appears to be an indispensable link in the activation of NOXs. Our data also suggested that suppressing p22phox could serve as a novel and straightforward therapeutic target for retinal disorders involving oxidative stress. The protective role of p22phox is also supported by several other studies. It has been reported that downregulation of p22phox in human lens epithelial B3 (HLEB3) cell decreased NOX activity, ROS generation, and cell proliferation^21^. AAV-mediated p22phox siRNA prominently suppressed the development of CNV in mice^20^. In this study, we focused on the potential protective role of downregulating p22phox in Ang II-induced inflammation accompanying oxidative stress. We found inhibition of NOX by downregulating p22phox exhibited a potent anti-inflammatory effect by virtue of reducing the expression of inflammatory cytokines.

Next, we explored the anti-inflammatory mechanisms of downregulating p22phox in Ang II-mediated inflammation. The MAPK signaling pathway, which includes JNK, p38 and ERK1/2 MAPKs, is strongly involved in oxidative stress and also plays an important role in inflammatory responses^24^,^51^. Both *in vitro* and *in vivo* studies have demonstrated that the phosphorylation level of p38 MAPK, ERK1/2 and JNK increased in response to Ang II^52^,^53^. In agreement with previous studies in other cellular systems^52^,^53^, we showed p38, ERK1/2 and JNK phosphorylation increased in ARPE-19 cells stimulated with Ang II. However, an *in vitro* study observed that JNK was not regulated by Ang II in ARPE-19 cells^54^. One reason for this discrepancy in JNK regulation could be the different incubation time used in these two studies. The ARPE-19 cells were stimulated by Ang II for only 5 minutes in the aforementioned study, and for 48 hours in our experiment. An *in vivo* study from the same team also supported this explanation. They found that phosphorylated ERK and p38 MAPK expression was increased in RPE from mice exposed to Ang II, which is in agreement with their results in ARPE-19 cells. In contrast, the level of phosphorylated JNK was dramatically increased in mice infused with Ang II^54^. The differential regulation of the JNK may be explained by the fact that RPE cells were exposed to Ang II for a much shorter time during the *in vitro* study, while mice were treated for several days *in vivo*. In addition, there is the additional possibility that a higher concentration of Ang II treatment may activate JNK.

We observed that the phosphorylation levels of p38 MAPK, ERK1/2 and JNK were increased in response to Ang II in ARPE-19 cell. Our results were consistent with previous findings that inhibiting p38 MAPK, ERK1/2 or JNK with their specific inhibitors decreased the production of inflammatory cytokines including IL-8, MCP-1 and IL-6. Our results indicated that all of the three MAPK family members were involved in the process of the Ang II-induced inflammatory response in ARPE-19 cells. It has been reported that the expression of p22phox increases in response to Ang II^55^. We investigated whether the anti-inflammatory effect of downregulating p22phox was associated with the MAPK pathway. Our data showed that downregulating p22phox dramatically inhibitd the phosphorylation of p38 MAPK, ERK1/2 and JNK.

Activation of NF-κB signaling is involved in the inflammation response to Ang II. Ang II influences NF-κB activation, stimulating nuclear translocation of the p65 subunit and degradation of transcription inhibition factor-κB (IκB)^56^. ROS also activates NF-κB, which plays a central and crucial role in inducing the expression of inflammatory cytokines^57^. We found that p22phox siRNA transfection not only decreased Ang II-induced expression of IL-8, MCP-1 and IL-6 but also reduced the degradation of IκBα and the nuclear translocation of NF-κB subunit p65.

Taken together, our results provides the proof-of-concept that downregulating p22phox is protective in oxidative stress accompanying ocular inflammation. They indicate that reducing p22phox levels evokes the inhibition of intracellular ROS generation, NOX activation, and inflammatory cytokine expression during ocular inflammation. Downregulation of p22phox may be closely correlated with the suppression of the MAPK and NF-κB pathways. To the best of our knowledge, our study is the first to highlight the importance and mechanism of downregulating p22phox as an inflammation-suppressor in ocular oxidative stress. Hence, we propose that downregulating p22phox could break the vicious cycle between oxidative damage and inflammation. This manipulation could be an effective and protective strategy against various retinal diseases. Our study has revealed that p22phox could serve as a potential therapeutic target and might be a promising marker for gene therapy in retinal diseases. However, further research should be conducted to investigate the protective role of p22phox downregulation in different ocular disease models that share similar pathological processes involving oxidative stress and inflammation, such as diabetic retinopathy, retinopathy of prematurity (ROP), glaucoma and uveitis. To design an optimal therapeutic strategy and avoid undesirable side effects, it is also important to investigate the long-term effects of suppressing p22phox in animal models.

In summary, the present research demonstrates that downregulating p22phox results in an anti-inflammatory effect in Ang II-induced oxidative stress in ARPE-19 and hRPE cells. Inhibiting the MAPK and NF-κB pathways is involved in this beneficial effect. These results support the belief that suppressing p22phox may break the vicious cycle between oxidative stress and inflammation in numerous ocular diseases and that reducing p22phox is a promising therapeutic target.

## Methods

### Cell culture

A human retinal pigment epithelial cell line, ARPE-19 was obtained from the American Type Culture Collection (ATCC, Manassas, VA). The cells were cultured in Dulbecco’s modified Eagle’s medium F-12 nutrient mixture (DMEM/F-12, Invitrogen, Carlsbad, CA) with 10% fetal bovine serum (FBS, Invitrogen, Carlsbad, CA), 100 U/ml penicillin, and 100 μg/ml streptomycin. The cells were incubated in a humidified incubator at 37 °C in 5% CO_2_. The cells were passaged every 5 to 7 days. Trypsin-EDTA (Invitrogen, Carlsbad, CA) was used to detach the cells after reaching confluence. Cells were then diluted to 1:3 or 1:4. Cells between passages 19 and 25 were used for experiments.

Human primary retinal pigment epithelia (hRPE) were obtained from donors’ eyes from the Chongqing Eye Bank. This study was approved by the Ethics Committee of the First Affiliated Hospital of Chongqing Medical University, Chongqing, China. The methods were carried out in accordance with the approved guidelines and regulations. The hRPE was prepared and cultured as follows: after the vitreous and retina were removed, hRPE cells were harvested by digestion with 0.05% trypsin and 0.02% EDTA, and washed two times with Hank’s balanced salt solution (HBSS) (GE Healthcare Life Sciences, USA). The cells were suspended in complete Dulbecco’s modified Eagle’s medium F-12 nutrient mixture (DMEM/F-12, Invitrogen, Carlsbad, CA) containing 10% fetal bovine serum, 100 U/ml penicillin and 100 μg/ml streptomycin. Cells were incubated in a humidified incubator at 37 °C in 5% CO_2_.

### Cell viability assay

The Cell Counting Kit-8 (CCK-8) assay (Sigma-Aldrich, St. Louis, MO) was used to investigate the effects of Angiotensin II (Ang II) on the viability of ARPE-19 cells. WST-8 [2-(2-methoxy-4-nitrophenyl)-3-(4-nitrophenyl)-5-(2, 4-disulfophenyl)-2H-tetrazolium, monosodium salt] is a highly water-soluble tetrazolium salt. It can be reduced by a dehydrogenase and converted to a yellow colored formazan dye in cells, which is soluble in the culture media. The amount of formazan dye is directly proportional to the number of living cells. ARPE-19 cells were plated in 96-well plates at a density of 1 × 10^4^ cells per well. After the cells became confluent, 100 μl of serum-free medium was replaced to starve the cells for 24 hours. Ang II was added to the wells at final concentrations of 10^−4^, 10^−5^, 10^−6^, 10^−7^, 10^−8^ M and cultured for 24, 48 and 72 hours. Next, 10 μl of WST-8 was added to each well. The optical density was read at 450 nm using a microplate reader (Molecular Devices, Sunnyvale, CA). Cells cultured without Ang II were used as a control.

### Annexin-V FITC/Propidium Iodide (PI) apoptosis assay

Briefly, 2 × 10^5^ of ARPE-19 cells were seeded into 24-well plates. When the cells became confluent, 1 ml of serum-free medium was replaced to starve the cells for 24 hours. Ang II was added to the wells at final concentrations of 10^−4^, 10^−5^, 10^−6^, 10^−7^, 10^−8^ M and cultured for 24, 48 and 72 hours. Next, cells were trypsinized, washed twice with ice-cold PBS, and the supernatant was discarded. This assay was carried out following the manufacturer’s instructions from the Annexin-V FITC/PI apoptosis detection kit (Vazyme Biotech Co., Ltd., Nanjing, China). The cell pellets were resuspended with 100 μl of 1X binding buffer. Next, 5 μl of Annexin-V FITC and 5 μl of PI were added to the suspension and incubated at room temperature for 10 minutes in the dark. Subsequently, 400 μl of 1X binding buffer was added followed by measurement using flow cytometry (FCM, FACSAire, Becton Dickinson, USA).

### p22phox small-interfering RNA (siRNA) transfection and Angiotesin II (Ang II) treatment

Two different siRNAs targeting specific sequences of p22phox (NM_000101.1) and a negative control of scrambled siRNA not homologous to any gene, were synthesized by GenePharma (Shanghai, China). The sequences of these siRNAs are shown in Table 2. ARPE-19 cells and hRPE were seeded in 24-well plates (1-2 × 10^5^ cells/well) and cultured in 500 μl DMEM culture medium containing 10% FBS without antibiotics until the cells were 50%–60% confluent. Before transfection, cells were serum starved for 12 hours. Lipofectamine® 2000 transfection reagent (Invitrogen, Carlsbad, CA) was used to perform the transfection. The siRNA-complex was pre-mixed according to the manufacturer’s instructions and added to the 24-well plates. The final concentration of p22phox and scrambled siRNA was 100 nM per well. Six hours after transfection, the medium was changed to fresh serum-free culture medium without antibiotics. Next, Ang II (10^−6^ M, Sigma-Aldrich, St. Louis, MO) was added to stimulate the cells for 48 hours, and cell samples were collected for subsequent assays.

### Real-Time PCR analysis

Total RNA was extracted from the cells using TRIzol reagent (Invitrogen, Carlsbad, CA) according to the manufacturer’s instructions. Complementary DNA (cDNA) was generated using the PrimeScript^®^ RT reagent kit (Takara Biotechnology, Dalian, China). Real-time PCR was performed according to the manufacturer’s instruction with the ABI Prism 7500 system (Applied Biosystems, Foster City, CA). Each reaction was run in duplicate. Relative quantification was achieved by the comparative 2^−ΔΔCt^ method as described in our previous study^58^. The sequences of PCR primer pairs are shown in Table S1. The entire list of 9 primers can be found as Supplementary Table S1 online. Real-time PCR was performed in a volume of 20 μl using SYBR^®^ Premix Ex Taq^TM^ II (Takara Biotechnology, Dalian, China). The conditions were 95 °C for 10 min, followed by 40 cycles of 15 s at 95 °C and 60 s at 60 °C.

### Flow cytometry analysis for intracellular reactive oxygen species (ROS)

Production of reactive oxygen species (ROS) was detected using the non-fluorescent probe 2′,7′-dichlorofluorescein diacetate (DCFH-DA). DCFH-DA diffused into cells and hydrolyzed into the 2′,7′-dichlorofluorescein (DCFH) which was hard to pass through the cytomembrane. Then DCFH reacted with ROS to form the fluorescent product DCF. Briefly, ARPE-19 cells and hRPE were transfected with p22phox siRNA and stimulated with Ang II as aforementioned. Then the medium was removed and cells were washed with ice-cold PBS. DCFH-DA (Beyotime, Shanghai, China) was diluted in fresh DMEM/F12 at a final concentration of 10 μM and incubated with cells for 30 min at 37 °C. After DCFH-DA treatment, the chemicals were removed and loaded cells were washed three times with PBS. The fluorescence was read at 488 nm excitation and 525 nm emission by the BD FACS Vantage SE Flow Cytometer (BD, Shanghai, China).

### Western blotting analysis

Cells were rinsed with ice-cold PBS and lysed by Radio Immuno Precipitation Assay (RIPA) Lysis Buffer (Beyotime, Shanghai, China) including 1% protease inhibitor (Beyotime, Shanghai, China). The cell lysate was centrifuged, and the supernatant was collected. The protein concentration was determined with a bicinchoninic acid (BCA) protein kit (Beyotime, Shanghai, China). All samples were diluted in SDS loading buffer (Beyotime, Shanghai, China) and boiled for 5 minutes. Equal amounts of protein (80 μg) were loaded on a 10% polyacrylamide gel for SDS-PAGE electrophoresis. The protein was then electroblotted onto nitrocellulose membranes (Millipore, Billerica, MA). Membranes were blocked with 5% skim milk or 5% bovine serum albumin (BSA) and incubated with specific primary antibodies against p22phox (1:200, Santa Cruz Biotechnology, Inc., Santa Cruz, CA), p65 (1:1200, ABCAM, Cambridge, MA), β-actin (1:100, ABCAM, Cambridge, MA), p38, ERK1/2, phosphorylated p38 (p-p38), phosphorylated ERK1/2 (p-ERK1/2) (1:1000, Cell Signaling Technology, Danvers, MA), JNK, and phosphorylated JNK (p-JNK) (1:500, Cell Signaling Technology, Danvers, MA) overnight at 4 °C, followed by secondary antibody incubation (1:3000, ABCAM, Cambridge, MA) at 37 °C for 1 hour. The membranes were further developed with a Western Bright^TM^ ECL kit (Advansta, Menlo Park, CA). Bands were analyzed using ImageJ software (Version 1.43, Broken Symmetry Software, Bethesda, MD). Analysis was normalized against the housekeeping protein β-actin. Band intensities of p-p38 MAPK, p-ERK1/2 and p-JNK were normalized to p38 MAPK, ERK1/2 and JNK, respectively.

### The signal transduction mechanism analysis with inhibitors of p38 MAPK, ERK1/2, JNK and NF-κB after stimulation with Ang II

We investigated whether the MAPK and NF-κB signal pathways were involved in Ang II–induced cytokine expression. ARPE-19 cells were seeded in 24-well plates to become confluent. After being serum starved for 24 hours, cells were pre-incubated with p38MAPK inhibitor SB203580 (Cell Signaling Technology, Danvers, MA), JNK inhibitor SP600125 (Cell Signaling Technology, Danvers, MA), ERK1/2 inhibitor PD98059 (Cell Signaling Technology, Danvers, MA) and NF-κB inhibitor BAY11-7082 (Sigma-Aldrich, St. Louis, MO) at the concentration of 10 μM for 30 minutes. Subsequently, Ang II (10^−6^ M) was added to the medium. After 48 hours of incubation, the supernatants were collected and stored at −80 °C until analysis. Total RNA of the cells after treatment was extracted for further assay.

### Enzyme-linked immunosorbent assay (ELISA)

The supernatants of the cells treated with MAPK, NF-κB inhibitors followed by stimulating with Ang II were collected for detecting the concentrations of IL-6, IL-8 and MCP-1 by the human ELISA development kits (Duoset; R&D Systems, Minneapolis, MN) according to the manufacturer’s instructions.

### Statistical analysis

All results were expressed as mean ± SEM. Statistical analysis was performed with the GraphPad Prism 5 software (GraphPad Software, Inc., San Diego, CA). Experimental data were analyzed by one-way ANOVA followed by Bonferroni correction for multiple comparisons. *p* < 0.05 was considered to be significantly different.

## Additional Information

**How to cite this article**: Qiu, Y. *et al.* Downregulating p22phox ameliorates inflammatory response in Angiotensin II induced oxidative stress by regulating MAPK and NF-κB pathways in ARPE-19 cells. *Sci. Rep.* **5**, 14362; doi: 10.1038/srep14362 (2015).

## Supplementary Material

Supplementary Table S1

Supplementary Figure S1

## Figures and Tables

**Table 1:** Fold changes of mRNA and protein expression (VS Ang II group).

**group**	**cytokines**	**mRNA fold change**	**protein fold change**
Ang II+SB	IL-8	1.7	3.4
	MCP-1	2.5	1.8
	IL-6	3.5	5.2
Ang II+PD	IL-8	1.6	3.7
	MCP-1	3.2	1.8
	IL-6	2.0	2.5
Ang II+SP	IL-8	2.0	6.2
	MCP-1	2.6	3.1
	IL-6	1.9	1.6
Ang II+BAY	IL-8	3.7	3.0
	MCP-1	3.7	1.9
	IL-6	2.0	2.0

**Table 2:** Sequences of the siRNA.

**siRNA**	**Forward**	**Reverse**
p22phox siRNA-1	5′-GAAGGGCUCCACCAUGGAGTT-3′	5′-UCCAUGGUGGAGCCCUUCTT-3′
p22phox siRNA-2	5′-UUACUAUGUUCGGGCCGUCTT-3′	5′-GACGGCCCGAACAUAGUAATT-3′
scrambled siRNA	5′-UUCUCCGAACGUGUCACGUTT-3′	5′-ACGUGACACGUUCGGAGAATT-3′
