# Supplementary material for: Downregulating p22phox ameliorates inflammatory response in Angiotensin II-induced oxidative stress by regulating MAPK and NF-κB pathways in ARPE-19 cells
Source: Sci Rep. 2015 Sep 29;5:14362. doi: 10.1038/srep14362 (PMC4586461; doi:10.1038/srep14362)
Supplement: Supplementary Table S1 [file srep14362-s1.pdf]

Downregulating p22phox ameliorates inflammatory response in Angiotensin II-induced oxidative stress by regulating MAPK and NF-κB pathways in ARPE-19 cells

Yiguo Qiu[1], Lifei Tao[1], Chunyan Lei[1], Jiaming Wang[1], Peizeng Yang[1], Qiuhong Li[2], Bo Lei[1]

Table S1. Sequences of the primers

Primer	Forward	Reverse
P22phox	5'-AACGAGCAGGCGCTGGCGTCCG-3'	5'-GCTTGGGCTCGATGGGCGTCCACT-3'
MCP-1	5'-CTCATAGCAGCCACCTTCATTC-3'	5'-TCACAGCTTCTTTGGGACACTT-3'
IL-8	5'-GACATACTCCAAACCTTTCCACCC-3'	5'-CCAGACAGAGCTCTCTTCCATCAG-3'
IL-6	5'-AGTGAGGAACAAGCCAGAGC-3'	5'-CAGGGGTGGTTATTGCATCT-3'
NOX-1	5'-TTCACCAATTCCCAGGATTGAAGTGGATGGTC-3'	5'-GACCTGTCACGATGTCAGTGGCCTTGTCAA-3'
NOX-2	5'-AGAGGGTTGGAGGTGGAGAATT-3'	5'-GCACAAGGAGCAGGACTAGATGA-3'
NOX4	5'-CTGGAGGAGCTGGCTCGCCAACGAAG-3'	5'-GTGATCATGAGGAATAGCACCACCACCATGCAG-3'
IκBα	5‘-CGGACTGCCCTTCACCTC-3’	5‘-ACATCAGCCCCACACTTCAA-3’
β-actin	5'-GGATGCAGAAGGAGATCACTG-3'	5'-CGATCCACACGGAGTACTTG-3'
