# Supplementary material for: Downregulating p22phox ameliorates inflammatory response in Angiotensin II-induced oxidative stress by regulating MAPK and NF-κB pathways in ARPE-19 cells
Source: Sci Rep. 2015 Sep 29;5:14362. doi: 10.1038/srep14362 (PMC4586461; doi:10.1038/srep14362)
Supplement: Supplementary Figure S1 [file srep14362-s2.pdf]

Downregulating p22phox ameliorates inflammatory response in Angiotensin II-induced oxidative stress by regulating MAPK and NF-κB pathways in ARPE-19 cells

Yiguo Qiu[1], Lifei Tao[1], Chunyan Lei[1], Jiaming Wang[1], Peizeng Yang[1], Qiuhong Li[2], Bo Lei[1]

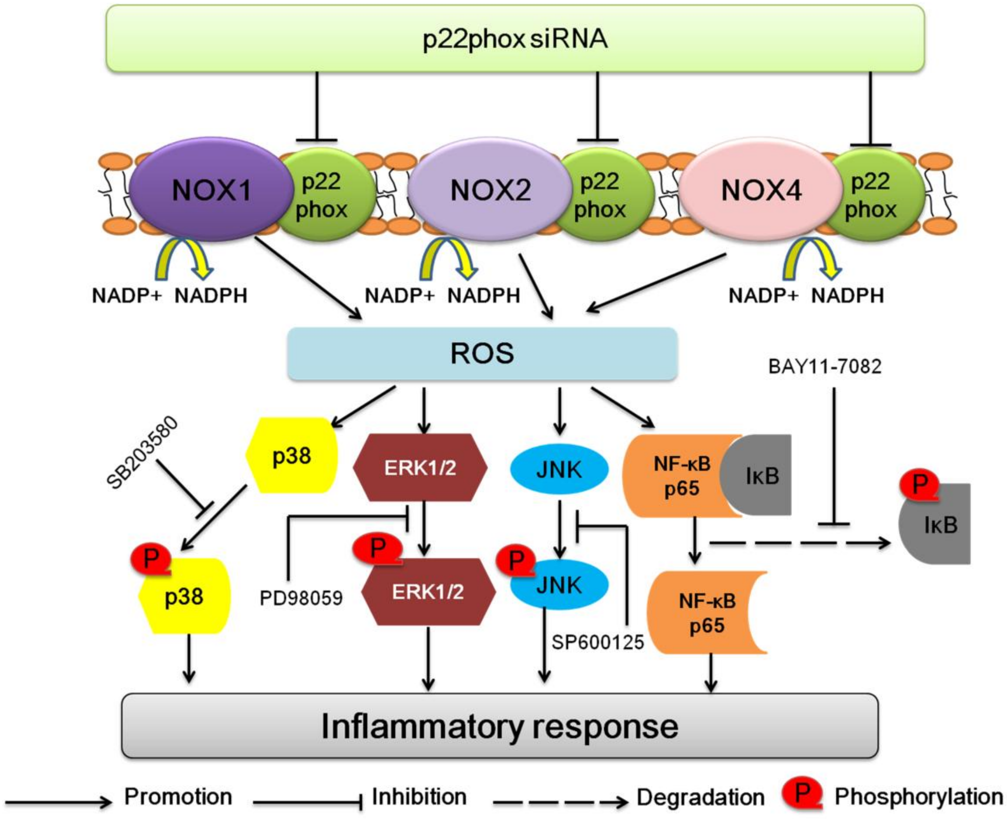

Supplementary Figure S1. The schematic diagram of the anti-inflammatory effect played by downregulating p22phox. Downregulating p22phox inhibits the activation of NOXs and the generation of intracellular ROS. The p38MAPK, ERK1/2, JNK and NF-κB pathways are involved in the anti-inflammatory effect.
